# 1,3-Bis(3,5-dimethyl­phen­yl)-5-methyl­benzene

**DOI:** 10.1107/S1600536810024013

**Published:** 2010-06-26

**Authors:** Ke-Wei Lei, Dong-Guo Xia, Jie Li

**Affiliations:** a State Key Laboratory Base of Novel Functional Materials and Preparation Science, Institute of Solid Materials Chemistry, Faculty of Materials Science and Chemical Engineering, Ningbo University, Ningbo 315211, People’s Republic of China

## Abstract

In the title compound, C_23_H_24_, the dihedral angles formed by the central benzene ring with the peripheral benzene rings are 29.90 (5) and 34.95 (5)°. The crystal packing is stabilized by π–π stacking inter­actions with centroid–centroid distances of 3.815 (4) Å.

## Related literature

For the role of terphenyls in organic synthesis, see: Wright & Vinod (2003 ▶). For conformational studies on terphenyls, see: Amorim da Costa *et al.* (1997 ▶).
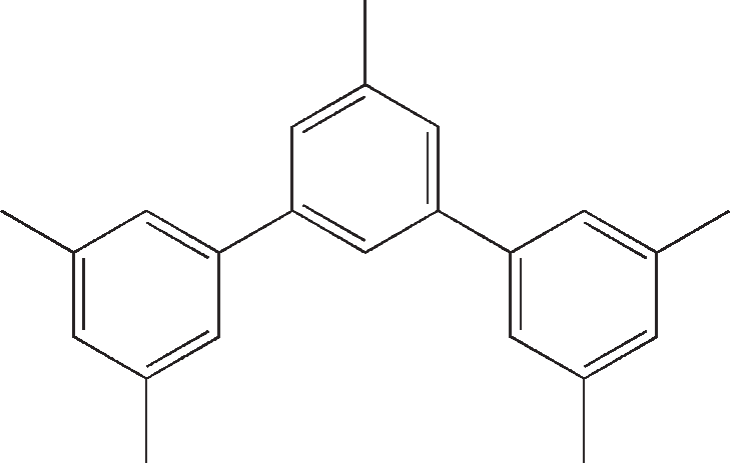


## Experimental

### 

#### Crystal data

C_23_H_24_
*M* *_r_* = 300.42
Monoclinic, 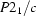

*a* = 14.955 (2) Å
*b* = 7.6081 (12) Å
*c* = 16.207 (2) Å
β = 106.839 (2)°
*V* = 1765.0 (5) Å^3^
*Z* = 4
Mo *K*α radiation
μ = 0.06 mm^−1^
*T* = 296 K
0.48 × 0.42 × 0.37 mm

#### Data collection

Bruker SMART APEXII diffractometer
Absorption correction: multi-scan (*SADABS*; Sheldrick, 2000 ▶) *T*_min_ = 0.970, *T*_max_ = 0.977
12155 measured reflections
3087 independent reflections
2485 reflections with *I* > 2σ(*I*)
*R*_int_ = 0.030

#### Refinement

*R*[*F* ^2^ > 2σ(*F* ^2^)] = 0.044
*wR*(*F* ^2^) = 0.133
*S* = 1.04
3087 reflections
213 parameters
H-atom parameters constrained
Δρ_max_ = 0.26 e Å^−3^
Δρ_min_ = −0.19 e Å^−3^

### 

Data collection: *APEX2* (Bruker, 2007 ▶); cell refinement: *SAINT* (Bruker, 2007 ▶); data reduction: *SAINT*; program(s) used to solve structure: *SHELXS97* (Sheldrick, 2008 ▶); program(s) used to refine structure: *SHELXL97* (Sheldrick, 2008 ▶); molecular graphics: *SHELXTL* (Sheldrick, 2008 ▶); software used to prepare material for publication: *SHELXTL*.

## Supplementary Material

Crystal structure: contains datablocks global, I. DOI: 10.1107/S1600536810024013/rz2458sup1.cif

Structure factors: contains datablocks I. DOI: 10.1107/S1600536810024013/rz2458Isup2.hkl

Additional supplementary materials:  crystallographic information; 3D view; checkCIF report

## Figures and Tables

**Fig. 1.**
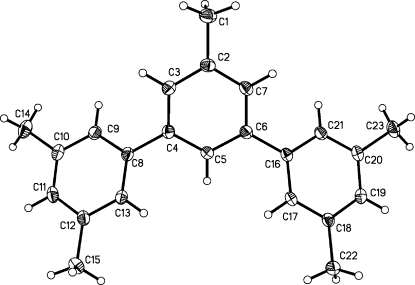
The structure of the title compound, showing 30% probability displacement ellipsoids and the atom-numbering scheme.

**Fig. 2.**
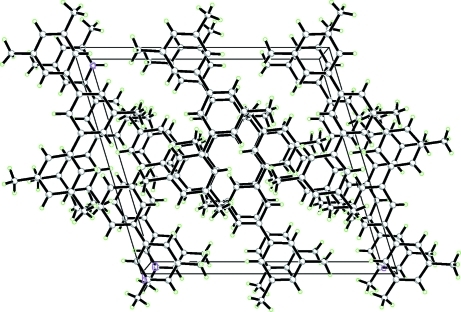
Crystal packing of the title compound viewed along the b axis.

## supplementary crystallographic information

### Comment

Terphenyl compounds play an important role in organic synthesis and as heat exchanger, due to the possibility of a methyl group on the terphenyl core to be oxidized to carboxylic acid (Wright & Vinod, 2003).The conformation of *m*-terphenyls has been studied under a variety of conditions; because these molecules are formed by three phenyl rings connected by two C—C bonds, characteristic conformational changes occur with the rotations around the C—C bonds (Amorim da Costa *et al.*, 1997). Herein we report the synthesis and crystal structure of a new terphenyl compound.

The molecular structure of the title compound is illustrated in Fig. 1. Bond lengths and bond angles are within normal ranges.The dihedral angle formed by the peripheral C8–C13 and C16–C21 benzene rings with the central C2–C7 benzene ring are 34.95 (5) and 29.90 (5)° respectively. In the crystal packing,the centroid-to centroid distance of 3.815 (4)Å between the planes of adjacent C8–C13 benzene rings suggests that the molecules are engaged in offset face-to-face π-π stacking interactions (Fig. 2).

### Experimental

1,3-Dibromo-5-methylbenzene (88.1 mmol, 22.02 g), 3,5-dimethylphenylboronic acid (211.6 mmol, 31.73 g) and triphenylphosphine (17.62 mmol, 4.62 g) were dissolved in 1,2-dimethoxyethane (120 ml), the 240 ml of a 2*M* K_2_CO_3_ (480 mmol) aqueous solution were added and the mixture was purged with nitrogen. Palladium acetate (0.988 g, 0.025 eq.) was added and the mixture was refluxed for 18 h. The two phases were then separated and the aqueous phase was extracted with ethyl acetate (3 *x* 250 ml). The combined organic phases were washed with water (250 ml) and dried over MgSO_4_. After evaporation of the solvent, the oily residue was purified by bulb-to-bulb distillation to afford the crude title compound. Recrystallization from ethyl acetate gave colourless crystal after 3 days. Yield: 78.9%. Calcd. for C_23_H_24_: C, 92.01%; H, 7.99%; Found: C, 91.87%; H, 8.08%.

### Refinement

All H atoms were placed in geometrically idealized positions and treated as riding on their parent atoms, with C—H = 0.93-0.96 Å, and with *U*_iso_(H) = 1.2*U*_eq_(C) or 1.5*U*_eq_(C) for methyl H atoms.

### Crystal data

**Table:**

C_23_H_24_	*F*(000) = 648
*M**_r_* = 300.42	*D*_x_ = 1.131 Mg m^−^^3^
Monoclinic, *P*2_1_/*c*	Mo *K*α radiation, λ = 0.71073 Å
Hall symbol: -P 2ybc	Cell parameters from 4929 reflections
*a* = 14.955 (2) Å	θ = 2.6–27.5°
*b* = 7.6081 (12) Å	µ = 0.06 mm^−^^1^
*c* = 16.207 (2) Å	*T* = 296 K
β = 106.839 (2)°	Block, colourless
*V* = 1765.0 (5) Å^3^	0.48 × 0.42 × 0.37 mm
*Z* = 4	

### Data collection

**Table:**

Bruker SMART APEXII diffractometer	3087 independent reflections
Radiation source: fine-focus sealed tube	2485 reflections with *I* > 2σ(*I*)
graphite	*R*_int_ = 0.030
phi and ω scans	θ_max_ = 25.0°, θ_min_ = 2.6°
Absorption correction: multi-scan (*SADABS*; Sheldrick, 2000)	*h* = −17→15
*T*_min_ = 0.970, *T*_max_ = 0.977	*k* = −9→9
12155 measured reflections	*l* = −19→19

### Refinement

**Table:**

Refinement on *F*^2^	Primary atom site location: structure-invariant direct methods
Least-squares matrix: full	Secondary atom site location: difference Fourier map
*R*[*F*^2^ > 2σ(*F*^2^)] = 0.044	Hydrogen site location: inferred from neighbouring sites
*wR*(*F*^2^) = 0.133	H-atom parameters constrained
*S* = 1.04	*w* = 1/[σ^2^(*F*_o_^2^) + (0.0676*P*)^2^ + 0.6595*P*] where *P* = (*F*_o_^2^ + 2*F*_c_^2^)/3
3087 reflections	(Δ/σ)_max_ < 0.001
213 parameters	Δρ_max_ = 0.26 e Å^−^^3^
0 restraints	Δρ_min_ = −0.19 e Å^−^^3^

### Special details

**Table:**

Geometry. All e.s.d.'s (except the e.s.d. in the dihedral angle between two l.s. planes) are estimated using the full covariance matrix. The cell e.s.d.'s are taken into account individually in the estimation of e.s.d.'s in distances, angles and torsion angles; correlations between e.s.d.'s in cell parameters are only used when they are defined by crystal symmetry. An approximate (isotropic) treatment of cell e.s.d.'s is used for estimating e.s.d.'s involving l.s. planes.
Refinement. Refinement of *F*^2^ against ALL reflections. The weighted *R*-factor *wR* and goodness of fit *S* are based on *F*^2^, conventional *R*-factors *R* are based on *F*, with *F* set to zero for negative *F*^2^. The threshold expression of *F*^2^ > σ(*F*^2^) is used only for calculating *R*-factors(gt) *etc*. and is not relevant to the choice of reflections for refinement. *R*-factors based on *F*^2^ are statistically about twice as large as those based on *F*, and *R*- factors based on ALL data will be even larger.

### Fractional atomic coordinates and isotropic or equivalent isotropic displacement parameters (Å^2^)

**Table:**

	*x*	*y*	*z*	*U*_iso_*/*U*_eq_	
C1	0.28892 (13)	0.9286 (3)	−0.18056 (11)	0.0430 (5)	
H1A	0.3479	0.9757	−0.1822	0.064*	
H1B	0.2406	1.0136	−0.2032	0.064*	
H1C	0.2756	0.8235	−0.2147	0.064*	
C2	0.29293 (12)	0.8859 (2)	−0.08860 (10)	0.0323 (4)	
C3	0.21125 (11)	0.8739 (2)	−0.06402 (10)	0.0317 (4)	
H3A	0.1544	0.9012	−0.1039	0.038*	
C4	0.21242 (11)	0.8221 (2)	0.01890 (10)	0.0293 (4)	
C5	0.29861 (11)	0.7810 (2)	0.07781 (10)	0.0289 (4)	
H5A	0.3004	0.7432	0.1328	0.035*	
C6	0.38219 (11)	0.7953 (2)	0.05594 (10)	0.0292 (4)	
C7	0.37750 (11)	0.8499 (2)	−0.02774 (10)	0.0325 (4)	
H7A	0.4326	0.8623	−0.0429	0.039*	
C8	0.12366 (11)	0.8067 (2)	0.04287 (10)	0.0290 (4)	
C9	0.04176 (11)	0.7501 (2)	−0.01771 (10)	0.0332 (4)	
H9A	0.0432	0.7229	−0.0733	0.040*	
C10	−0.04181 (11)	0.7333 (2)	0.00311 (11)	0.0357 (4)	
C11	−0.04208 (11)	0.7735 (2)	0.08674 (11)	0.0357 (4)	
H11A	−0.0972	0.7609	0.1017	0.043*	
C12	0.03733 (11)	0.8317 (2)	0.14839 (11)	0.0329 (4)	
C13	0.12016 (11)	0.8473 (2)	0.12570 (10)	0.0300 (4)	
H13A	0.1740	0.8856	0.1667	0.036*	
C14	−0.12950 (12)	0.6743 (3)	−0.06379 (13)	0.0489 (5)	
H14A	−0.1131	0.6047	−0.1066	0.073*	
H14B	−0.1645	0.7753	−0.0906	0.073*	
H14C	−0.1668	0.6052	−0.0367	0.073*	
C15	0.03426 (13)	0.8813 (3)	0.23773 (12)	0.0474 (5)	
H15A	0.0840	0.8231	0.2798	0.071*	
H15B	−0.0246	0.8462	0.2451	0.071*	
H15C	0.0414	1.0063	0.2451	0.071*	
C16	0.47339 (11)	0.7497 (2)	0.11903 (10)	0.0286 (4)	
C17	0.48839 (11)	0.7751 (2)	0.20766 (10)	0.0294 (4)	
H17A	0.4406	0.8217	0.2270	0.035*	
C18	0.57282 (11)	0.7322 (2)	0.26727 (10)	0.0302 (4)	
C19	0.64405 (11)	0.6637 (2)	0.23696 (11)	0.0321 (4)	
H19A	0.7006	0.6331	0.2763	0.039*	
C20	0.63239 (11)	0.6404 (2)	0.14940 (11)	0.0318 (4)	
C21	0.54670 (11)	0.6821 (2)	0.09147 (10)	0.0310 (4)	
H21A	0.5379	0.6646	0.0329	0.037*	
C22	0.58804 (12)	0.7640 (3)	0.36219 (11)	0.0404 (4)	
H22A	0.5334	0.8179	0.3708	0.061*	
H22B	0.6408	0.8403	0.3837	0.061*	
H22C	0.5996	0.6541	0.3925	0.061*	
C23	0.71275 (12)	0.5809 (3)	0.11702 (12)	0.0437 (5)	
H23A	0.7562	0.5144	0.1612	0.066*	
H23B	0.7437	0.6818	0.1026	0.066*	
H23C	0.6893	0.5088	0.0668	0.066*	

### Atomic displacement parameters (Å^2^)

**Table:**

	*U*^11^	*U*^22^	*U*^33^	*U*^12^	*U*^13^	*U*^23^
C1	0.0487 (11)	0.0466 (11)	0.0349 (9)	−0.0097 (9)	0.0141 (8)	0.0031 (8)
C2	0.0378 (9)	0.0300 (8)	0.0302 (8)	−0.0042 (7)	0.0115 (7)	−0.0003 (7)
C3	0.0308 (9)	0.0306 (8)	0.0315 (8)	−0.0005 (7)	0.0057 (7)	0.0018 (7)
C4	0.0273 (8)	0.0286 (8)	0.0319 (8)	−0.0007 (6)	0.0086 (7)	−0.0009 (7)
C5	0.0276 (8)	0.0325 (8)	0.0279 (8)	−0.0004 (7)	0.0097 (7)	0.0009 (6)
C6	0.0283 (8)	0.0296 (8)	0.0311 (8)	−0.0014 (6)	0.0107 (7)	−0.0030 (7)
C7	0.0313 (9)	0.0340 (9)	0.0355 (9)	−0.0052 (7)	0.0150 (7)	−0.0025 (7)
C8	0.0249 (8)	0.0290 (8)	0.0321 (8)	0.0024 (6)	0.0065 (7)	0.0045 (6)
C9	0.0288 (9)	0.0364 (9)	0.0321 (9)	0.0005 (7)	0.0054 (7)	0.0021 (7)
C10	0.0264 (9)	0.0331 (9)	0.0432 (10)	0.0004 (7)	0.0033 (7)	0.0064 (7)
C11	0.0237 (8)	0.0372 (9)	0.0482 (10)	0.0023 (7)	0.0135 (7)	0.0081 (8)
C12	0.0286 (8)	0.0336 (9)	0.0381 (9)	0.0046 (7)	0.0121 (7)	0.0058 (7)
C13	0.0236 (8)	0.0322 (9)	0.0324 (8)	0.0005 (6)	0.0054 (6)	0.0022 (7)
C14	0.0313 (10)	0.0518 (12)	0.0549 (12)	−0.0053 (8)	−0.0013 (8)	0.0052 (9)
C15	0.0397 (10)	0.0610 (13)	0.0465 (11)	0.0018 (9)	0.0208 (9)	−0.0012 (9)
C16	0.0245 (8)	0.0300 (8)	0.0333 (8)	−0.0033 (6)	0.0118 (7)	−0.0002 (7)
C17	0.0253 (8)	0.0317 (8)	0.0349 (9)	−0.0007 (6)	0.0148 (7)	0.0000 (7)
C18	0.0274 (8)	0.0311 (8)	0.0336 (9)	−0.0031 (6)	0.0110 (7)	0.0016 (7)
C19	0.0244 (8)	0.0316 (9)	0.0398 (9)	−0.0001 (6)	0.0084 (7)	0.0040 (7)
C20	0.0263 (8)	0.0280 (8)	0.0445 (10)	−0.0019 (6)	0.0155 (7)	−0.0024 (7)
C21	0.0289 (8)	0.0340 (9)	0.0331 (8)	−0.0042 (7)	0.0138 (7)	−0.0043 (7)
C22	0.0355 (10)	0.0521 (11)	0.0337 (9)	0.0029 (8)	0.0104 (8)	0.0038 (8)
C23	0.0314 (9)	0.0490 (11)	0.0554 (11)	0.0032 (8)	0.0198 (8)	−0.0073 (9)

### Geometric parameters (Å, °)

**Table:**

C1—C2	1.509 (2)	C13—H13A	0.9300
C1—H1A	0.9600	C14—H14A	0.9600
C1—H1B	0.9600	C14—H14B	0.9600
C1—H1C	0.9600	C14—H14C	0.9600
C2—C7	1.387 (2)	C15—H15A	0.9600
C2—C3	1.393 (2)	C15—H15B	0.9600
C3—C4	1.396 (2)	C15—H15C	0.9600
C3—H3A	0.9300	C16—C21	1.397 (2)
C4—C5	1.399 (2)	C16—C17	1.401 (2)
C4—C8	1.492 (2)	C17—C18	1.388 (2)
C5—C6	1.399 (2)	C17—H17A	0.9300
C5—H5A	0.9300	C18—C19	1.397 (2)
C6—C7	1.401 (2)	C18—C22	1.508 (2)
C6—C16	1.489 (2)	C19—C20	1.390 (2)
C7—H7A	0.9300	C19—H19A	0.9300
C8—C13	1.393 (2)	C20—C21	1.388 (2)
C8—C9	1.398 (2)	C20—C23	1.513 (2)
C9—C10	1.392 (2)	C21—H21A	0.9300
C9—H9A	0.9300	C22—H22A	0.9600
C10—C11	1.391 (2)	C22—H22B	0.9600
C10—C14	1.508 (2)	C22—H22C	0.9600
C11—C12	1.385 (2)	C23—H23A	0.9600
C11—H11A	0.9300	C23—H23B	0.9600
C12—C13	1.396 (2)	C23—H23C	0.9600
C12—C15	1.510 (2)		
			
C2—C1—H1A	109.5	C10—C14—H14A	109.5
C2—C1—H1B	109.5	C10—C14—H14B	109.5
H1A—C1—H1B	109.5	H14A—C14—H14B	109.5
C2—C1—H1C	109.5	C10—C14—H14C	109.5
H1A—C1—H1C	109.5	H14A—C14—H14C	109.5
H1B—C1—H1C	109.5	H14B—C14—H14C	109.5
C7—C2—C3	118.56 (14)	C12—C15—H15A	109.5
C7—C2—C1	120.78 (15)	C12—C15—H15B	109.5
C3—C2—C1	120.60 (15)	H15A—C15—H15B	109.5
C2—C3—C4	121.76 (15)	C12—C15—H15C	109.5
C2—C3—H3A	119.1	H15A—C15—H15C	109.5
C4—C3—H3A	119.1	H15B—C15—H15C	109.5
C3—C4—C5	118.18 (14)	C21—C16—C17	118.10 (14)
C3—C4—C8	120.59 (14)	C21—C16—C6	120.92 (14)
C5—C4—C8	121.21 (14)	C17—C16—C6	120.97 (14)
C6—C5—C4	121.59 (14)	C18—C17—C16	121.62 (14)
C6—C5—H5A	119.2	C18—C17—H17A	119.2
C4—C5—H5A	119.2	C16—C17—H17A	119.2
C5—C6—C7	118.08 (14)	C17—C18—C19	118.39 (15)
C5—C6—C16	121.04 (14)	C17—C18—C22	120.69 (14)
C7—C6—C16	120.86 (14)	C19—C18—C22	120.90 (15)
C2—C7—C6	121.78 (14)	C20—C19—C18	121.59 (15)
C2—C7—H7A	119.1	C20—C19—H19A	119.2
C6—C7—H7A	119.1	C18—C19—H19A	119.2
C13—C8—C9	118.38 (14)	C21—C20—C19	118.64 (14)
C13—C8—C4	121.21 (14)	C21—C20—C23	120.27 (15)
C9—C8—C4	120.41 (14)	C19—C20—C23	121.00 (15)
C10—C9—C8	121.59 (15)	C20—C21—C16	121.63 (15)
C10—C9—H9A	119.2	C20—C21—H21A	119.2
C8—C9—H9A	119.2	C16—C21—H21A	119.2
C11—C10—C9	118.20 (15)	C18—C22—H22A	109.5
C11—C10—C14	121.26 (16)	C18—C22—H22B	109.5
C9—C10—C14	120.54 (16)	H22A—C22—H22B	109.5
C12—C11—C10	121.97 (15)	C18—C22—H22C	109.5
C12—C11—H11A	119.0	H22A—C22—H22C	109.5
C10—C11—H11A	119.0	H22B—C22—H22C	109.5
C11—C12—C13	118.58 (15)	C20—C23—H23A	109.5
C11—C12—C15	120.93 (15)	C20—C23—H23B	109.5
C13—C12—C15	120.48 (15)	H23A—C23—H23B	109.5
C8—C13—C12	121.27 (15)	C20—C23—H23C	109.5
C8—C13—H13A	119.4	H23A—C23—H23C	109.5
C12—C13—H13A	119.4	H23B—C23—H23C	109.5
			
C7—C2—C3—C4	−1.9 (2)	C10—C11—C12—C13	−1.1 (2)
C1—C2—C3—C4	175.21 (15)	C10—C11—C12—C15	177.55 (17)
C2—C3—C4—C5	−0.3 (2)	C9—C8—C13—C12	0.2 (2)
C2—C3—C4—C8	−178.69 (15)	C4—C8—C13—C12	−179.50 (15)
C3—C4—C5—C6	1.7 (2)	C11—C12—C13—C8	0.4 (2)
C8—C4—C5—C6	−179.86 (14)	C15—C12—C13—C8	−178.23 (16)
C4—C5—C6—C7	−1.0 (2)	C5—C6—C16—C21	148.88 (16)
C4—C5—C6—C16	−179.49 (14)	C7—C6—C16—C21	−29.6 (2)
C3—C2—C7—C6	2.6 (2)	C5—C6—C16—C17	−31.8 (2)
C1—C2—C7—C6	−174.43 (15)	C7—C6—C16—C17	149.78 (15)
C5—C6—C7—C2	−1.3 (2)	C21—C16—C17—C18	−1.0 (2)
C16—C6—C7—C2	177.26 (15)	C6—C16—C17—C18	179.61 (14)
C3—C4—C8—C13	−145.64 (16)	C16—C17—C18—C19	0.6 (2)
C5—C4—C8—C13	36.0 (2)	C16—C17—C18—C22	178.76 (15)
C3—C4—C8—C9	34.7 (2)	C17—C18—C19—C20	0.8 (2)
C5—C4—C8—C9	−143.73 (16)	C22—C18—C19—C20	−177.33 (15)
C13—C8—C9—C10	−0.2 (2)	C18—C19—C20—C21	−1.8 (2)
C4—C8—C9—C10	179.53 (15)	C18—C19—C20—C23	174.69 (16)
C8—C9—C10—C11	−0.5 (2)	C19—C20—C21—C16	1.3 (2)
C8—C9—C10—C14	179.17 (16)	C23—C20—C21—C16	−175.17 (16)
C9—C10—C11—C12	1.1 (2)	C17—C16—C21—C20	0.0 (2)
C14—C10—C11—C12	−178.51 (16)	C6—C16—C21—C20	179.43 (15)
